# Supplementary material for: Floria: fast and accurate strain haplotyping in metagenomes
Source: Bioinformatics. 2024 Jun 28;40(Suppl 1):i30–8. doi: 10.1093/bioinformatics/btae252 (PMC11211831; doi:10.1093/bioinformatics/btae252)
Supplement: btae252_Supplementary_Data [file btae252_supplementary_data.pdf]

Supplementary Materials for Floria

Algorithm 1: Floria phasing algorithm pseudocode

Input : BAM, Contig, VCF
Output: Haplosets $Hap_1, ..., Hap_\ell$ for Contig
$\epsilon \leftarrow$ Provided by user or estimated(BAM)
$\mathcal{R} = \{R, ...\} \leftarrow$ Break contig into chunks and let each $R \in \mathcal{R}$ be the set of reads mapped to a chunk
Partitions = {}
# parallel for loop below
for R *in* $\mathcal{R}$ **do**
  $MEC^* = []$; one-indexed array
  $Parts_R = []$; one-indexed array
  for $i = 1$ *to MaxPloidy* **do**
    $P \leftarrow$ BeamSearch(R,ϵ, i)
    $P \leftarrow$ IterativeOptimize(P,ϵ)
    $MEC^*[i] \leftarrow$ Getϵ−MECScore(P, ϵ)
    $Parts_R[i] \leftarrow P$
    if $MEC^*(i) <$ *NumberOfAlleles(R)* $\cdot\epsilon$ **then**
      $BestPart \leftarrow P$
      Break
    end
    $alpha(k) = 1/(k + 1/3)$
    if $MEC^*(i)/MEC^*(i-1) \geq \frac{1}{(1-\epsilon)(1+\alpha(i))}$ *and* $i \geq 2$ **then**
      $BestPart \leftarrow Parts_R[i-1]$
      Break
    end
    $BestPart \leftarrow P$
  end
  Partitions $\leftarrow$ Partitions $\cup \{BestPart\}$
end
$(V, E) \leftarrow$ Construct Weighted DAG By Shared Reads(Partitions)
$w \leftarrow$ Construct Flow Via Linear Program(V, E)
$Hap_1, \ldots, Hap_\ell$ = Get Paths With Max Min Flow Via DP(V, E, w)
Return $Hap_1, \ldots, Hap_\ell$

1 MEC score degeneracy and the epsilon-MEC score

In the beam search procedure, the initial starting partition is empty. This is an issue for MEC optimization because putting a read in an empty set is always the optimal greedy move as it does not increase the MEC score. We would prefer having similar reads cluster together even if one of the sets is empty. To combat this degeneracy, we define the epsilon-MEC score. Let $\epsilon - MEC(R_1, , R_k) = MEC(R_1, , R_k) + \epsilon \cdot S$ where S is the number of consensus alleles that only have a single read supporting it and ϵ is the previously defined error parameter. The ϵ-MEC score regularizes the degeneracy issue, as putting a read into an empty set increases the score by the expected number of errors in the read. We thus replace the MEC score in the beam phasing procedure with the ϵ-MEC score, using the ϵ-MEC score instead of MEC implicitly.

2 Final iterative optimization of partitions

Given the output of the beam search procedure for phasing, we further refine the partition by a Kernighan-Lin like algorithm [1]. For each read in each set of the partition, we check if moving the read to another set decreases the total epsilon-MEC score. If it does, we store

this move and the change in score. After checking all $(k-1)|R|$ possible moves, we execute either 1/10 of the best scoring moves or 1/3 of the best scoring moves if there are < 10 possible moves. We repeat this 10 times or until the epsilon-MEC score does not improve anymore.

3 Automatic parameter estimation of epsilon

Given that epsilon (ϵ) is an important parameter for both the ϵ-MEC score and beam search based phasing, we provide a heuristic to automatically estimate epsilon from data. From the input BAM file supplied by the user, we look at the pileup table for an arbitrary contig and obtain a distribution of the mismatch error rate. We then take the 66th percentile of the mismatch error rate as epsilon, which is slightly higher than the median estimate so as to account for reference bias and miscalled SNPs. We use this estimate at the start of Floria, and use it for all subsequent calculations.

The user can also provide a specified epsilon value if prior information is known about their sequencing technology. From testing, we recommend $\epsilon = 0.01$ for high-fidelity reads (e.g. Illumina or PacBio HiFi). For nanopore long-reads, we recommend ϵ from $0.01-0.06$ depending on the exact error rate; an exact value is hard to recommend due to the dependency of nanopore error rates on the base-calling method or nanopore chemistry. We analyzed the impact of epsilon on the three real-read communities discussed in the main text (Figure 2H) for various values of ϵ as well as comparing against our method of estimating ϵ (**Supplementary Figure 16**).

4 MEC score ploidy detection

Below we give a heuristic derivation of our local strain count (a.k.a "ploidy") detection algorithm, which is based on a simple probabilistic model with assumptions on the MEC score. While we use the ϵ-MEC score for phasing during the beam-search procedure, now we use the standard MEC score.

Suppose that we have reads from k-strains $R_1, ..., R_k$ and are trying to partition the $k-$strains into $n \leq k$ sets. We ask: what is the expected MEC score in this case?

We need some assumptions to make this tractable. Let L be the total number of SNPs across all strains. Let C_i be the average coverage of each strain (i.e. average coverage of each base in the strain) and let $C = \sum_i C_i$ with $C_1 > C_2 > C_3 ....$ We'll also assume that $C_i > \sum_{j>i} C_j$, i.e., the sum of the lowest j strains' abundances is less than the $j-1$th strain. While this assumption is not true in general, for the special case of two strains, it is almost always true.

Let's assume that the algorithm works by initially putting $R_1, ..., R_n$, the n most abundant strains, into separate sets in the partition and then lumping $R_{n+1}, ..., R_k$ into the set which minimizes the MEC. Let us assume that the reads call alleles with error rate ϵ. Then the expected MEC score is

$$\mathbb{E}[MEC(n,k)] \approx \sum_{i=1}^{n} \epsilon * C_i * L + \sum_{j=n+1}^{k} (1-\epsilon) * C_j * \min_{i=1,...,n} d(H(R_j), H(R_i)).$$

The first n terms are just allelic errors due to read errors. The second sum comes from lumping H_j into the set with H_i such that the distance between the two haplotypes is minimized; recall that $d(H(R_j), H(R_i))$ is the number of differing alleles between the two strains. The $1-\epsilon$ is the probability an allele is correctly called and thus the two haplotypes differ. There are also possible errors from the $n+1, ..., k$ strains actually having the same allele as strain i, but the alleles are miscalled – we ignore these errors and assume this is relatively small.

Note that we use the assumption $C_n > \sum_{j>n} C_j$ because implicit is the fact that even lumping all low abundance strains together does not change the consensus allele. If this abundance condition is not met, the above formula may still hold if the low abundance strains' reads are spread out over multiple haplosets and not lumped into a single one.

4.1 Ratio of MEC scores

We can use the above formula to answer the question: assume that we have n strains. What is the expected change in MEC score from an $n-1$ partition to an $n-$partition? The above formula gives:

$$\frac{\mathbb{E}[MEC(n,n)]}{\mathbb{E}[MEC(n-1,n)]} = \frac{\epsilon CL}{\sum_{i=1}^{n-1} \epsilon * C_i + (1-\epsilon) * C_n * \min_i d(H_n, H_i)}.$$

Rewriting this and letting $\min_i d(H_n, H_i) = d_n$, we get

$$= \frac{1}{(C - C_n)/C + (1-\epsilon)C_n/(C\epsilon) * (d_n/L)}.$$

Now we note don't want to detect strains that have too low coverage. We want the smallest covered strain to have at least ϵC coverage otherwise it is indistinguishable from errors. Thus we let $C_n \geq \epsilon C$. Plugging in this inequality yields

$$\geq \frac{1}{(1-\epsilon)(1 + d_n/L)}. \tag{1}$$

Thus equation 1 serves as a lower bound for what this ratio should be. Making an assumption that this upper bound $\geq$ is actually a reasonable approximation $\approx$, we can choose this value as a sort of decision boundary.

Intuitively, if the real MEC score has a smaller ratio than this decision boundary, the MEC score has decreased more than we expected, meaning that the new phasing is much better. If the MEC score has not decreased as much as we expected, there may only be $n-1$ strains in the phasing.

4.2 Algorithm

A simple algorithm to determine ploidy is to simply check $MEC(n,n)/MEC(n-1,n)$, the MEC scores from the beam-search phasing and see if it has a greater ratio than $\frac{1}{(1-\epsilon)(1+d_n/L)}$. If the real output ratio is greater, then we assume the ploidy is actually $n-1$, since it doesn't decrease as much as we expect. If the real output ratio is smaller than this conservative estimate, then we assume that n is a better estimate of the number of strains than $n-1$.

4.3 Empirical value for d_n/L

The value d_n/L represents the fraction of alleles that differ. Denote $d_n/L = \alpha_n$. While α_n depends on the exact strains considered, we can set it to an empirical function. Importantly, it should be decreasing as a function of n, because it is a minimum over n values. In floria v0.0.1, we have three possible functions for α_n corresponding to the option `--ploidy-sensitivity = 1,2,3`. The options 1,2,3 correspond to $\alpha_n = \frac{1}{n^a + b}$ where $a = 0.5, 1, 1$ and $b = 1, 1/3, 1$. As this parameter ranges from 1 to 3, α_n decreases and the required MEC ratio for stopping becomes larger; the algorithm clusters more aggressively. By default, we set the sensitivity to level 2; see **Supplementary Figure 16** for results for varying `--ploidy-sensitivity`.

5 HAPQ score

Spurious haplosets arising due to errors in our automatic clustering may occur. We seek to quantify haploset goodness by a measure we call HAPQ, analogous to MAPQ for read alignment. Like MAPQ, HAPQ is a measure of how likely it is that the haplotype arises due to error and should be filtered out.

Suppose we have haplosets Hap_1 and Hap_2, which are sets of reads. Let $C(Hap_1, Hap_2)$ be the interval containment of Hap_1 in Hap_2, meaning that if Hap_1 spans SNPs in $[a,b]$, and Hap_2 spans SNPs in $[c,d]$,

$$C(Hap_1, Hap_2) = \frac{|[a,b] \cap [c,d]|}{b - a + 1}$$

where a, b, c, d are SNP indices along the contig.

Let $s(Hap_1, Hap_2) = s(H(Hap_1), H(Hap_2))$ be the number of identical alleles shared between the two consensus haplotypes of the haplosets (i.e., the vartigs). Let L_B be the number of SNPs in the block, and $[a,b]$ defined as before for Hap_1. Then

$$\text{HAPQ}(Hap_1) = \min(60, t_1 * t_2 * t_3)$$

where

$$t_1 = \max_{Hap_i, i \neq 1} 40 \cdot \left[(1 - C(Hap_1, Hap_i)) \frac{s(Hap_1, Hap_i)}{s(Hap_1, Hap_i) + d(Hap1, Hapi)} \right],$$
$$t_2 = \min(1, |Hap_1|/3),$$
$$t_3 = \ln((b-a)/L_B + 1).$$

The term t_1 gives a low HAPQ if two haplosets look very similar to another haploset and one is highly contained in the other, meaning that this haploset should maybe be a part of

another haploset instead. t_2 makes sure there are enough reads in the haploset, and t_3 gives a high HAPQ if the vartig is long. Our formula is heuristic but is inspired by minimap2's formula for MAPQ [2], which is also a product of three terms with similar interpretations.

6 Floria strains count estimation

Assuming n contigs from a species, with size c and associated Floria's strain count s of minimum 1, we can define a species-level strain count (SSN) within a sample as the contigs size-adjusted mean of the strain counts:

$$\text{SNN}(Spe_1) = \frac{\sum_{i=1}^{n}(\max s_i, 1) * c_i}{\sum_{i=1}^{n}(c_i)}$$

To validate this estimator for different HAPQ values, we calculated the species strain counts for each species within the 40-species synthetic community and compared the value to the real strain numbers (**Supplementary Figure 17**). The estimation with $HAPQ > 15$ displays the most accurate result (Spearman correlation coefficient = 0.92), with a slight underestimation of the number of species for species with 2 or more strains, increasing with the number of strains. To avoid an overestimation of the strain count within real samples, we used a similar HAPQ threshold for the strain count estimation for the SPMP dataset (Figure 3). For the SPMP dataset analysis, SNN values for each species have been rounded to the nearest integer to obtain an approximated but more practical representation of the number of strains for each sample-species pair.

7 Spiked-in experiments

To assess the ability to identify the most abundant species within a sample with Kraken, we synthetically spiked-in 2 *Escherichia coli* strains within the SPMP *Escherichia* naturally depleted nanopore gut metagenome sample ERR7625355, based on Kraken taxonomic report results [3] Additionally, two more experiments with a strain from its sister species *Escherichia fergusonii* at different coverages (20X and 8X) were also produced to measure potential issues due to misidentification (**Supplementary Figure 1.A**). Three abundance measurements are calculated from the Kraken taxonomic reports: 1) The Kraken abundance corresponds to the abundance reported in the report, and 2) The assigned abundance is the sample normalized number of reads specifically assigned to a species (obtained with the Kraken –output file). For each species, the length of all assigned reads is then summed and divided by the reference genome length linked to the Kraken database to obtain 3) the theoretical coverage. Spiked-in experiments show the ability to obtain an acceptable estimate of both *E. coli* and *E. fergusonii* coverage with Kraken (**Supplementary Figure 1.B**) even at relatively low coverage. **Supplementary Figure 1.C** displays the number of species that would be retained for phasing with this procedure with the same ERR7625355 sample at various theoretical coverage thresholds.

8 Single-species communities design and construction

8.1 *K. pneumoniae* and *E.coli* synthetic-reads communities

The 3 *Klebsiella pneumoniae* community is composed of ASM1990034v1 (1st), ASM1990032v1 (2nd) and ASM1990030v1 (3rd). Nanopore reads were simulated using the same methods as for generating the synthetic gut metagenome; the 1st strain coverage has been fixed to 15X. The 7 *E. coli* strains community is constructed using the same strains as Figure 4 in the Strainberry paper [4], with 20X coverage for K-12 strain (used as reference) and 30X for others.

8.2 Communities with real reads

For the real-read communities, we first generated the true assemblies by assembling the isolate reads individually as follows. Reads were assembled by Flye (v. 2.9.2-b1786) with the nano-raw input types and default options. Assemblies were further polished with Medaka (v. 1.7.2) consensus method and model `'r941_min_hac_g507'`. Next, we created communities of 2, 3 or 4 strains by grouping the isolate reads. These were constructed by considering the following strain ordering: *E. coli*: [`'786605'`, `'899091'`, `'542093'`, `'623214'`], *B. licheniformis*: [`'N413_barcode11'`, `'N419_barcode16'`, `'N419_barcode20'`, `'N413_barcode03'`], *K. pneumoniae*: [`'N319_barcode04'`, `'N326_barcode18'`, `'N325_barcode01'`, `'N319_barcode12'`]. For less than 4 strains, strains used are picked from left to right (e.g: `'786605'`, `'542093'`

for a 2-strain community). Strain coverages were fixed at [25, 20, 15, 30] for four strains by subsampling reads, [25, 20, 15] for three strains, and so forth. Reads were provided to the Floria-PL subsampling pipeline (seed = 50) and phased with the same software versions used for the synthetic metagenome analysis. For each species, the reference used for reads mapping is the first one of the list (e.g. `'786605'`). For the main results, haplotigs were assembled using the Flye assembler.

9 Computational resources benchmarking

Benchmarking of all phasing software and major components of the phasing processes was based on snakemake benchmarking module results. We decided here to use a *CPU normalized wall time metric*, defined as wall time divided by the number of processors used as input to an overall pipeline, instead of the directly provided CPU time metric for benchmarking tool performance.

This decision was made based on three factors: First, phased assembly solutions tested here rely on multiple steps with inconsistent CPU usages, leading to intermittent and sometimes very long but low CPU loading (i.e, a period when the defined number of CPUs is underused) such as for Strainberry's variant calling step. Second, such pipelines are more and more run on cloud computing units for which one of the main factors of consideration is the instance lifespan. Last, we assume here that the instances system CPU usage is negligible compared to the high CPU usage of most of the phased assembly software.

All phased assembly results generated during the phasing assessment steps were done on a c6a12x AWS instance with the exception of StrainXPress for which a r5a12x was used to accommodate its high memory usage. Most of the multi-threaded steps were done with 48 CPUs, except when references were split and for which only 8 CPUs were used for each step. SPMP phasing has been processed on a separate cluster powered by Intel Xeon Platinum 8268 48-Cores 2.90 GHz processors with 192GB DDR4 2933MHz available.

10 Floria short-read phasing benchmark

While Floria produces long, accurate haplotypes for long-read data, many metagenomic samples only have short-read sequences available. Leveraging the same 120-strain synthetic community as before (section **Synthetic metagenome generation**), using ART[5] and we assessed Floria's short-read phasing and assembly against StrainXPress, a strain-level assembler, and the strain-oblivious assembler Megahit.

Floria assembles more strains (mean 72% aligned, **Supplementary Figure 13**) than Megahit (mean 50% aligned) but less than StrainXPress (mean 84% aligned). Nevertheless, this improvement for StrainXPress comes at the cost of a fragmented assembly with more than 6.9×105 sequences (against 3.5×105 and 1×105 for Floria and Megahit respectively) and a higher duplication ratio (1.32 and 1.18 for StrainXPress and Floria respectively). Furthermore, Floria requires drastically fewer computational resources than StrainXPress. Floria is more than 6 times faster than StrainXPress when considering variant calling, mapping, and haplotyping, and is more than 2 times faster when including base-level assembly after haplotyping (**Supplementary Figure 13**). StrainXPress takes more than 243 GB of memory compared to 29 GB for Floria, severely limiting its usage outside large computer clusters.

11 Reciprocal vartig mapping algorithm

For a pair of samples, we deduced corresponding vartigs between the two samples by the following reciprocal mapping procedure. First, for one of the samples which we call the "query" sample, we scan over each vartig in the query and find vartigs in the other "reference" sample for which this query vartig shares > 2 SNPs and has no errors – we call this a **hit**, giving us a set of hits for each query vartig. Second, we find a maximal sequence of non-overlapping hits subject to the following score:

$$Score(V_i, V_j) = s(V_i, V_j) - 0.2 * d(V_i, V_j).$$

Here V_i, V_j are the vartigs on the SNP-level, and the s, d functions are the same as before. For each query vartig, we get a sequence of hits with their respective scores. To get the maximal sequence of non-overlapping hits, we can use a standard dynamic programming. This returns a sequence of optimal hits for the query vartig to a set of reference vartigs. To get the final correspondence, we swap the query and reference and redo the procedure. We take these optimal hits that are present in both the original and swapped outputs to be our final correspondences.

Once we had our optimal reciprocal hits, we only retained hits with 0 errors, or with $< 95\%$ error rate (i.e. 1 differing SNP for every 20 SNPs) and used these hits to define strain-level vartig correspondences.

Our vartig aligner is available in the following repository: `https://github.com/bluenote-1577/vartig-utils`.

References

[1] B. W. Kernighan and S. Lin, "An efficient heuristic procedure for partitioning graphs," *The Bell System Technical Journal*, vol. 49, pp. 291–307, Feb. 1970. Conference Name: The Bell System Technical Journal.

[2] H. Li, "Minimap2: pairwise alignment for nucleotide sequences," *Bioinformatics*, vol. 34, pp. 3094–3100, Sept. 2018.

[3] J.-S. Gounot, C. Minghao, D. Bertrand, W.-Y. Saw, A. Ravikrishnan, A. Low, Y. Ding, N. H. Qi Amanda, L. W. Lin Tan, T. Yik-Ying, H. Seedorf, and N. Nagarajan, "Genome-centric analysis of short and long read metagenomes reveals uncharacterized microbiome diversity in Southeast Asians," preprint, Genomics, May 2022.

[4] R. Vicedomini, C. Quince, A. E. Darling, and R. Chikhi, "Strainberry: automated strain separation in low-complexity metagenomes using long reads," *Nature Communications*, vol. 12, p. 4485, July 2021. Bandiera_abtest: a Cc_license_type: cc_by Cg_type: Nature Research Journals Number: 1 Primary_atype: Research Publisher: Nature Publishing Group Subject_term: Bioinformatics;Genome assembly algorithms;Metagenomics;Software Subject_term_id: bioinformatics;genome-assembly-algorithms;metagenomics;software.

[5] W. Huang, L. Li, J. R. Myers, and G. T. Marth, "ART: a next-generation sequencing read simulator," *Bioinformatics*, vol. 28, pp. 593–594, Feb. 2012.

B

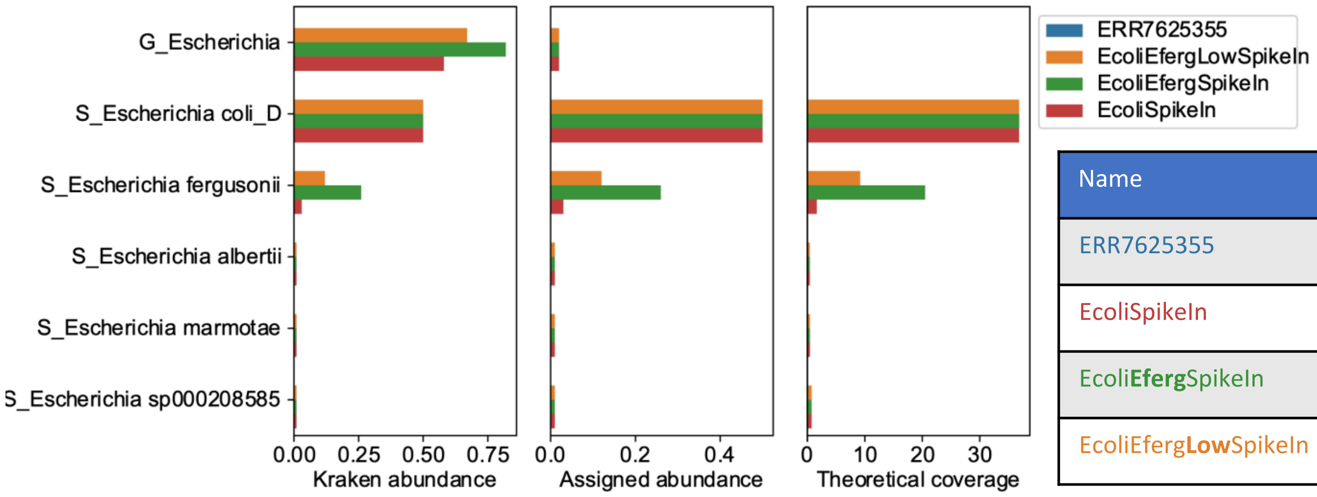

A

Name	E. coli strain #1	E. coli strain #2	E. fergusonii
ERR7625355	0	0	0
EcoliSpikeIn	20	30	0
Ecoli**Eferg**SpikeIn	20	30	20
EcoliEferg**Low**SpikeIn	20	30	8

C

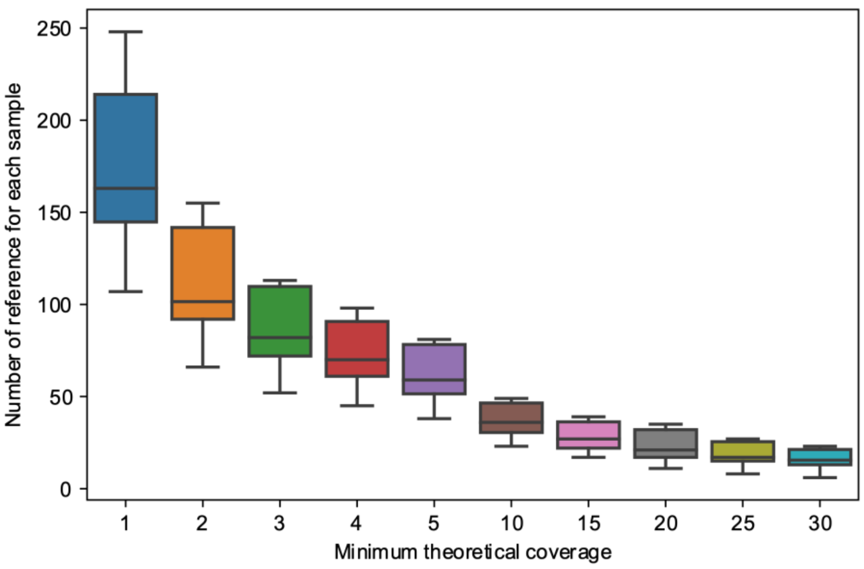

Supplemental Figure 1. SPMP sample spiked-in results to assess the kraken classification approach. A. Coverages of synthetic spiked-in species for each 3 experiments. ERR7625355 is the SPMP sample used for the spiked-in experiment. B. Spike-in results of *E. coli* and *E. fergusoni*. Kraken and assigned abundance, theoretical coverage for each Escherichia species for all 4 datasets described in [A]. C. Number of retained species from the Kraken classification approach based on different minimal theoretical coverage thresholds.

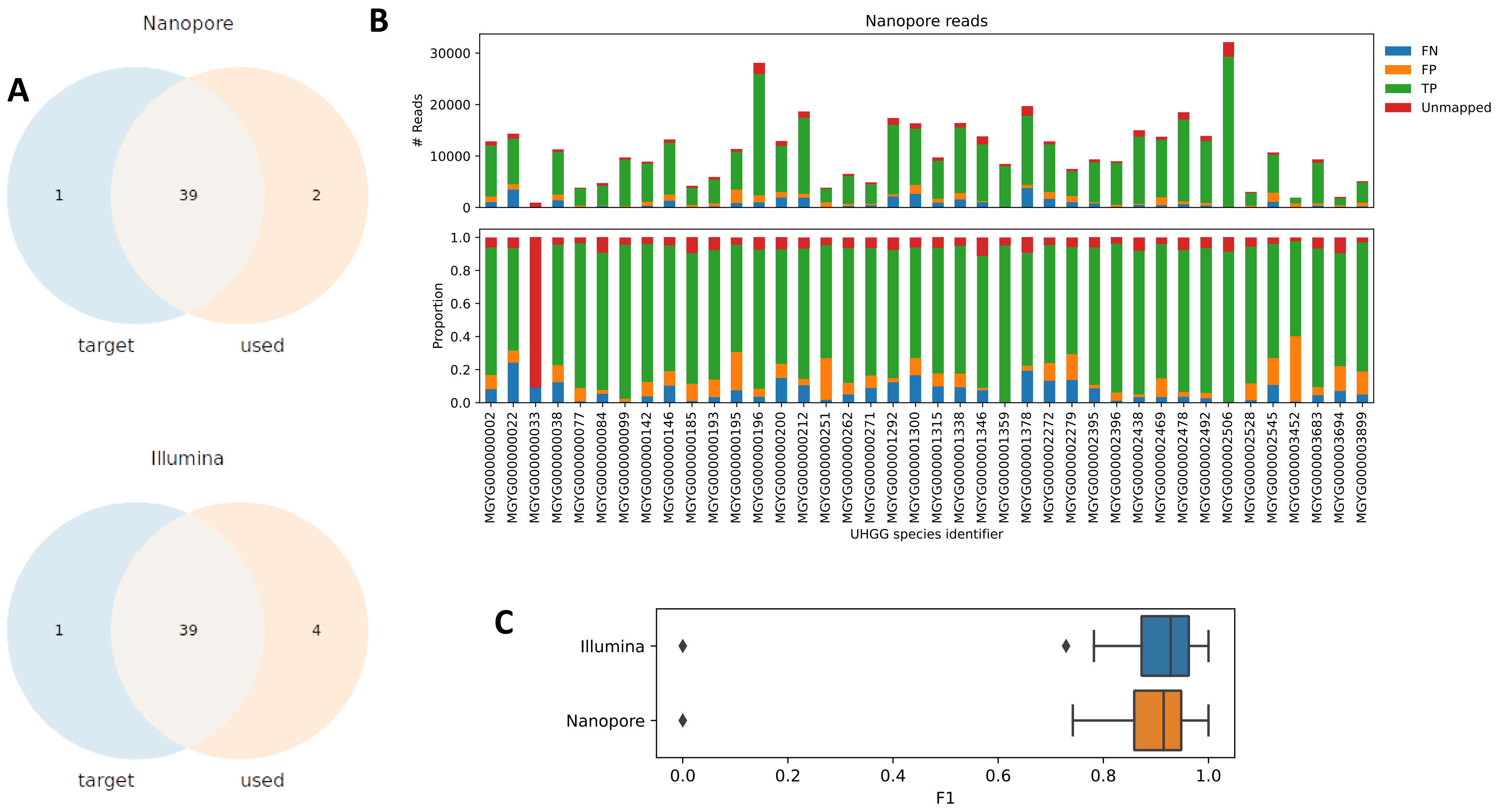

Supplementary Figure 2. Kraken mapping procedure on a 40-species, 120-strain metagenome. A. Venn diagram representing the intersection between the species that are in the synthetic metagenome (target) and species detected by the kraken procedure (used). **B.** Number of reads and their proportion for each reference genome. FP = mapped read comes from another species. TP = mapped read comes from the same species. FN = the species' read is mapped to another genome. C. Boxplot of the resulting F1 scores obtained with the above read mapping results.

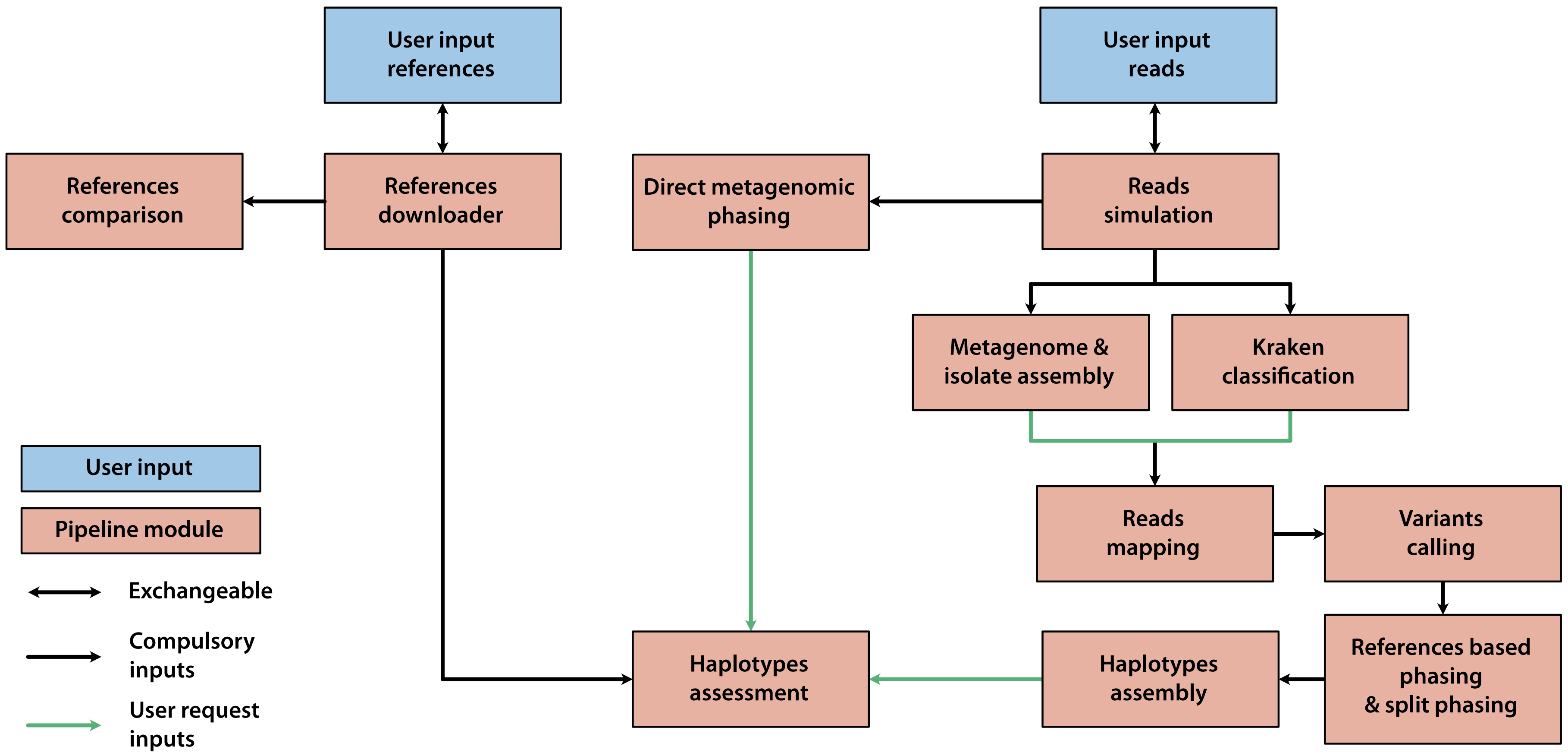

Supplementary Figure 3. Schematic representation of the benchmarking pipeline. Each orange/salmon block represents a module and blue blocks are user inputs. Reference genomes can either be provided by users or directly downloaded with the pipeline. Associated reads, either provided by users or simulated with the reference genomes, can be assembled to output phased contigs (StrainXpress, metaFlye), or processed through a read-mapping procedure and phasing (right, Floria, Strainberry or WhatsHap) and post-phasing haplotype assembly. Obtained haplotypes are finally compared to the references with the haplotype assessment module.

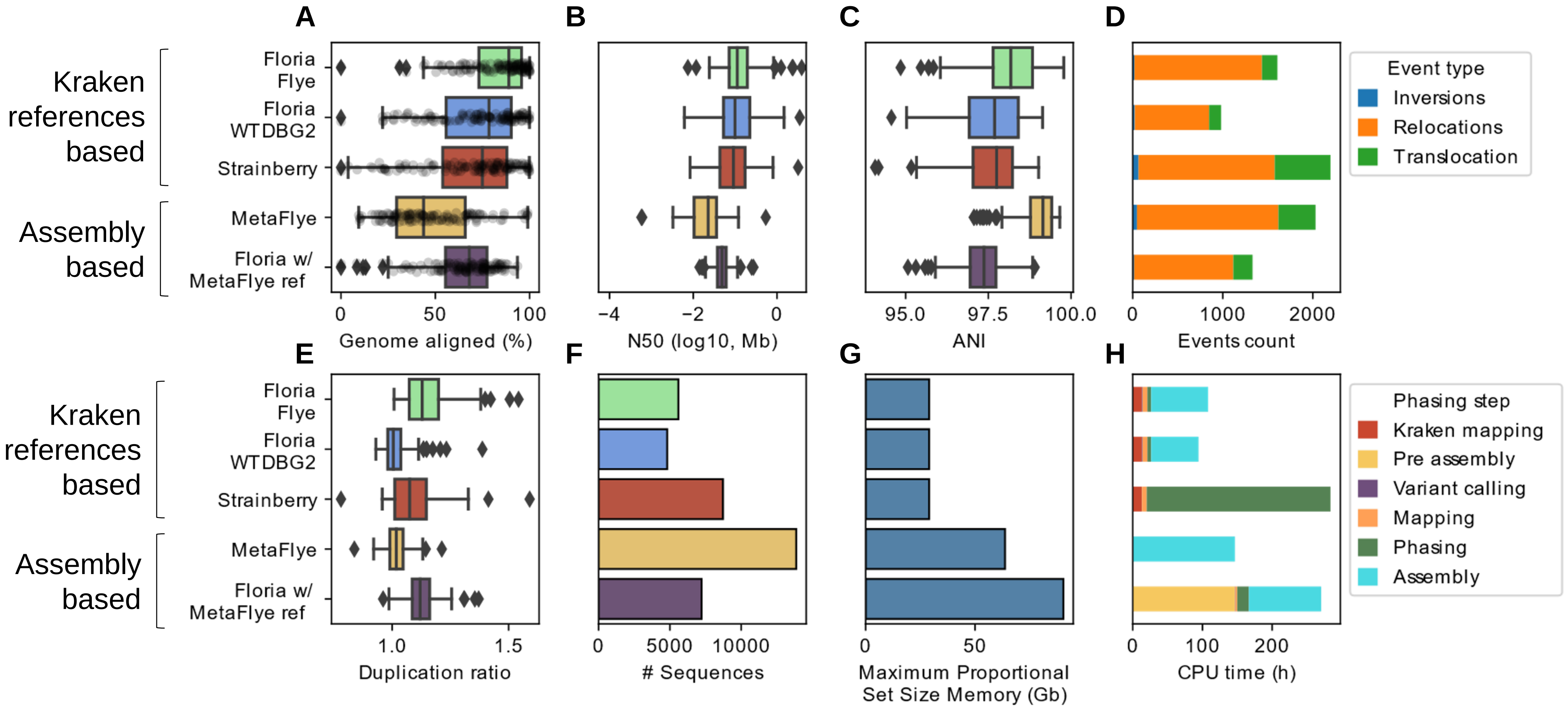

Supplementary Figure 4. Nanopore phasing assessment results (40 species, 120 strain community). Top left to bottom right: (A) Portion of strains genome aligned. (B) N50 of resulting assemblies. (C) ANI of each strain assembly vs the reference sequence of the strain (RSS). (D&E) Number of structural variants observed (D) and duplication ratio (E) of each phased assembly compared to their respective RSS. (F) Number of contigs obtained for each strain assembly. (G) Maximum memory size reached at any point of the pipeline for each phasing methods, adjusted (proportional) to consider shared library. (H) CPU time in hours for each phasing solutions at different steps. For Kraken references based, the assembler below Floria denotes which assembler has been used to assemble Floria haplotigs. Due to its global iterative process, Strainberry is unable to process metagenomic assemblies and is therefore restricted to a references-based approach.

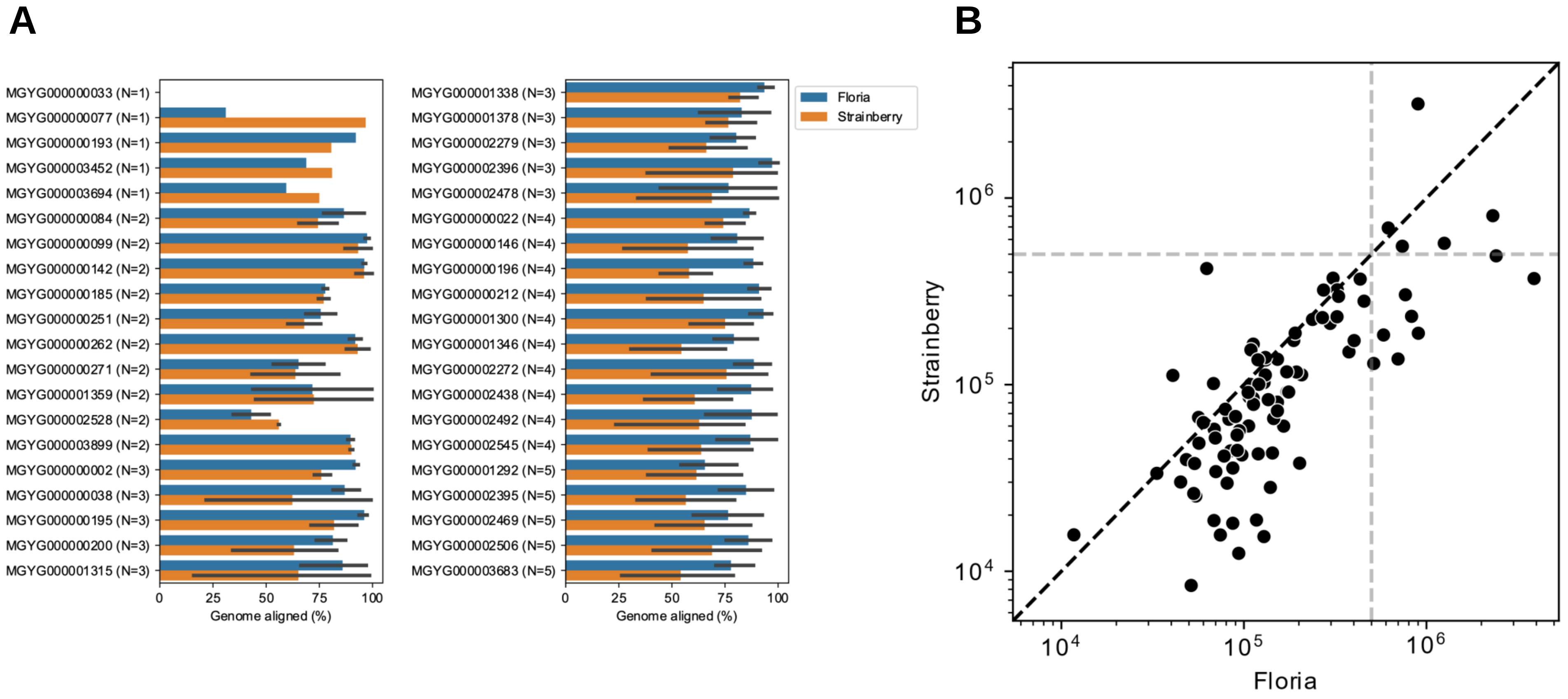

Supplementary Figure 5. Phasing statistics for the 40 species in the 120-strain synthetic metagenome. A. Barplot representation of the percentage of each strain's genome aligned for all 40 species, with N strains . When more than one strain is found within a species, bars represent the mean and additional black line is drawn representing a 95% confidential interval. B. Scatterplot representing the assembly N50 values (log scaled) for both Floria and Strainberry across all strains. Grey dashed lines delimit a 500Kb threshold. The black dashed line is the diagonal y = x.

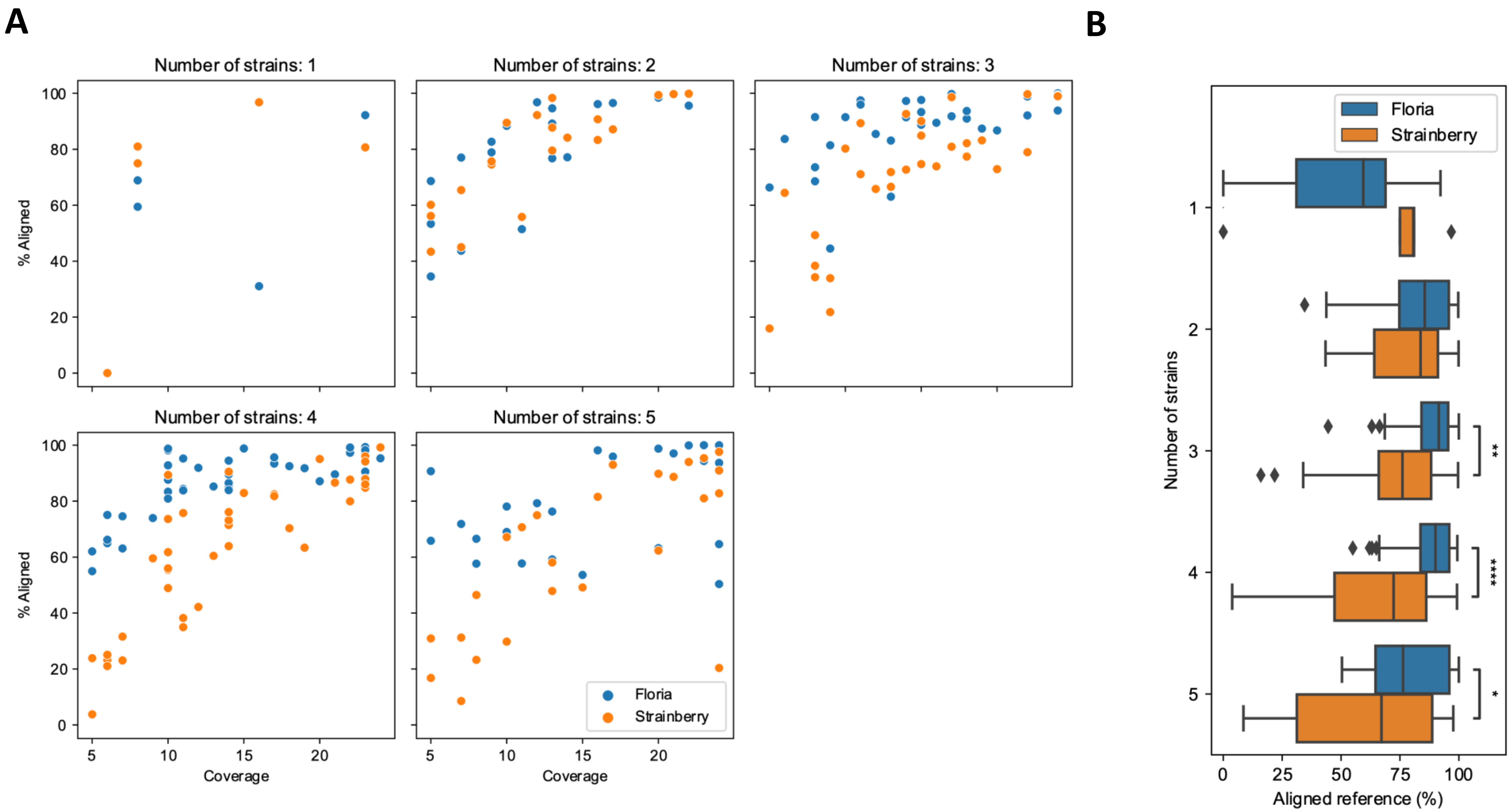

Supplementary Figure 6. Strains variations within the metagenomic synthetic community. A. Dot plots representing the percent of the strain reference aligned compared to the strain coverage, grouped by the number of strains used for each species. B. Boxplot representing % aligned reference similar to (A). Significant differences are represented by the * star symbols (Mann-Whitney-U test).

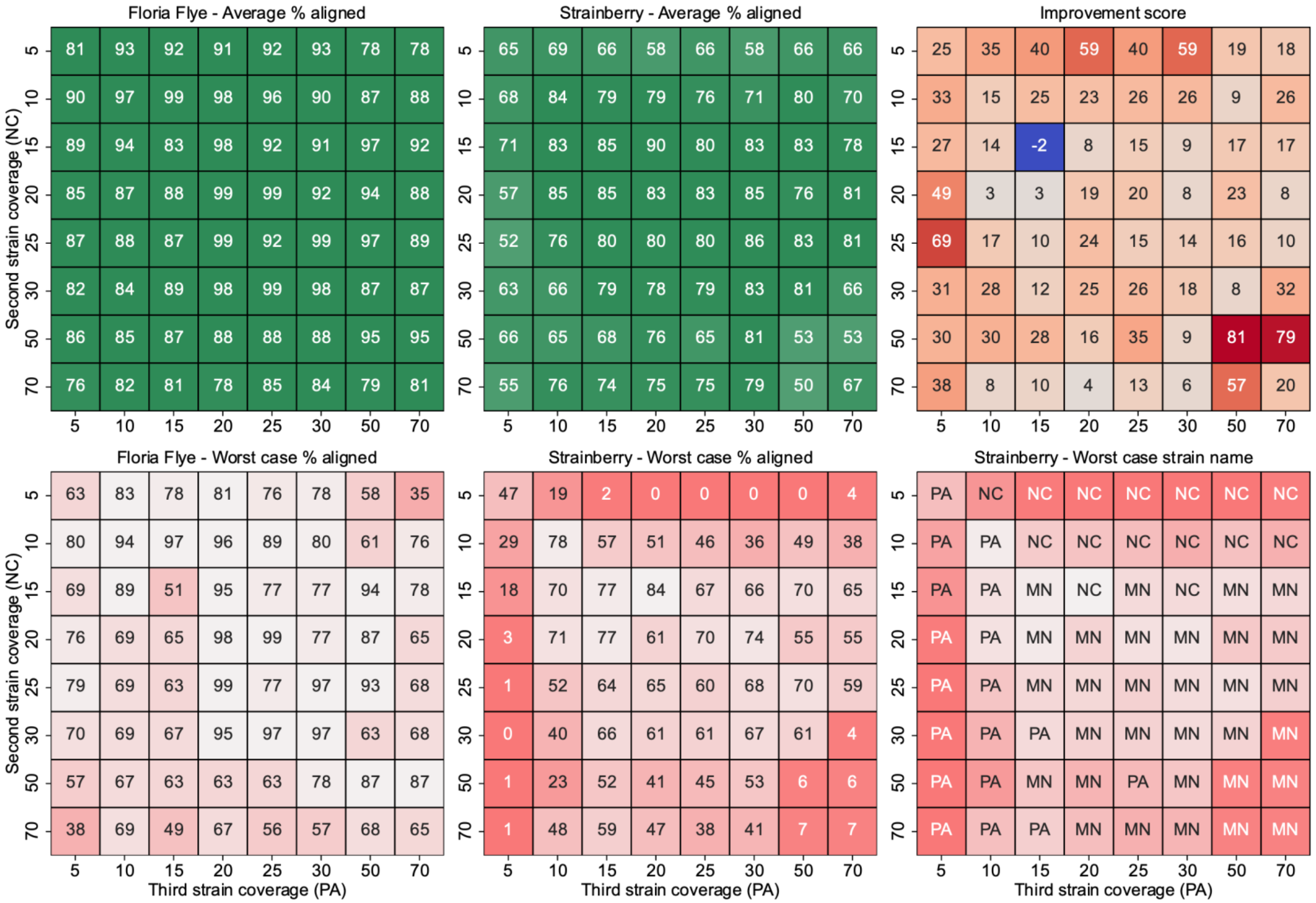

Supplementary Figure 7. Percent aligned metrics for various coverage combinations using a *K. pneumonia* three strains community. Metrics and related softwares are indicated in graph titles. Improvement score is calculated as (floria - strainberry) / strainberry x 100 using the percent aligned values. Worst case strain refers to the strains with the worst percent aligned. First strain coverage is 15X.

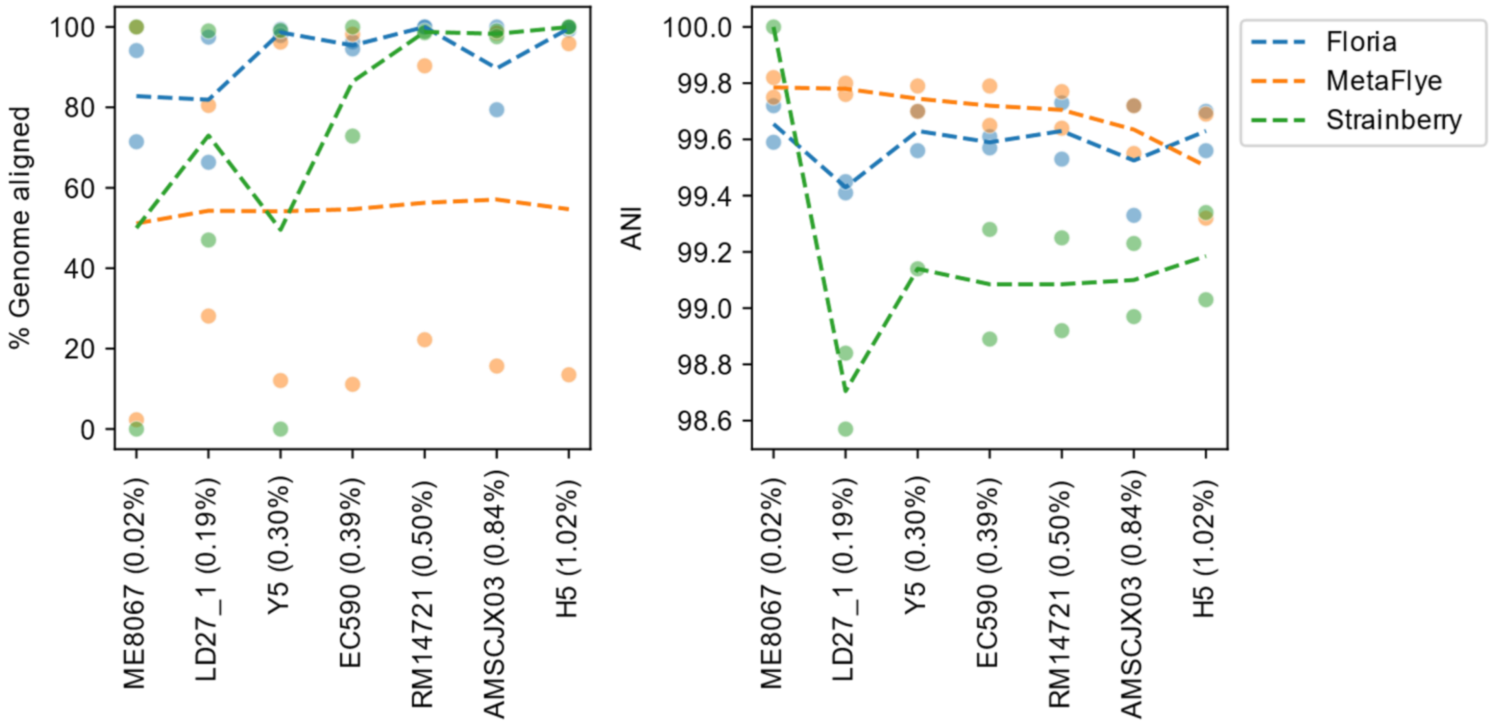

Supplementary Figure 8. Variable strain divergence. Percent genome aligned and ANI of multiple two-strain datasets consisting of simulated reads from two strains where one is *E. coli* strain K-12 and the other is another *E.* coli strain listed on the x-axis (with the divergence percentage shown in parenthesis). Dots represent the exact % aligned values (two dots are present if both strains are successfully resolved, otherwise single successfully resolved strain's dot is shown). Dashed lines represent the average value of the one or two strains.

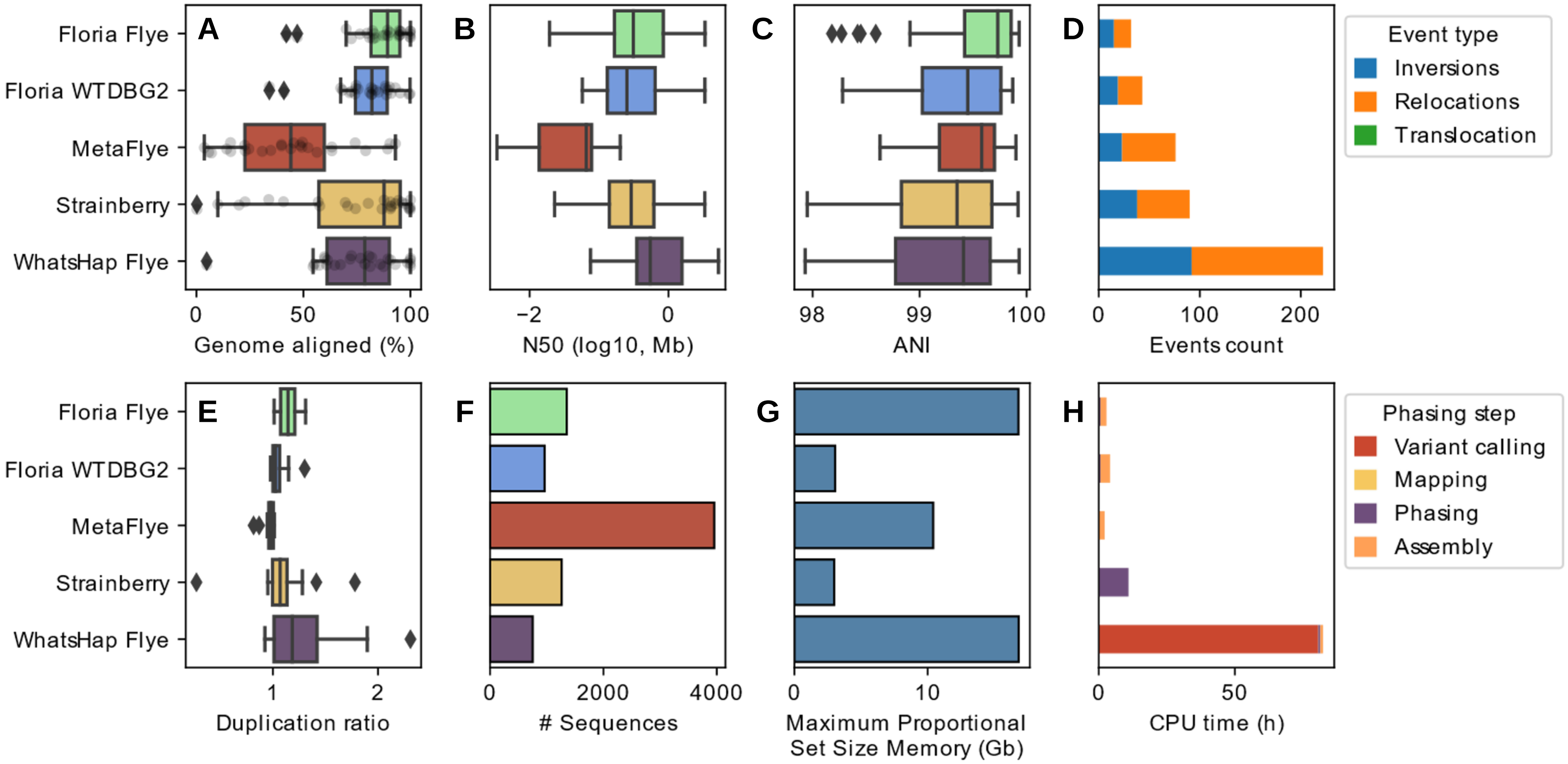

Supplementary Figure 9. **Real reads Nanopore phasing assessment results (2, 3 and 4 strain communities).** Average results for 2, 3 and 4-strain phasing of *B. lichenformis*, *E. coli* and *K. pneumoniae*. Top left to bottom right: (A) Percentage of strains' genomes aligned. (B) N50 of resulting assemblies. (C) ANI of each strain assembly vs. the reference sequence of the strain (RSS). (D&E) Number of structural variants observed (D) and duplication ratio (E) of each phased assembly compared to their respective RSS. (F) Number of contigs obtained for each strain assembly. (G) Maximum memory size reached at any point of the pipeline for each phasing method, adjusted (proportionally) to consider shared library. (H) CPU time in hours for each phasing solution at different steps. Longshot has been used for both Floria phasing results. WhatsHap requires a known ploidy, so we used freebayes with the true strain count as the input. For both WhatsHap and Floria, the haplotype assembler for the haplotype-specific reads is defined in the latter part of the label.

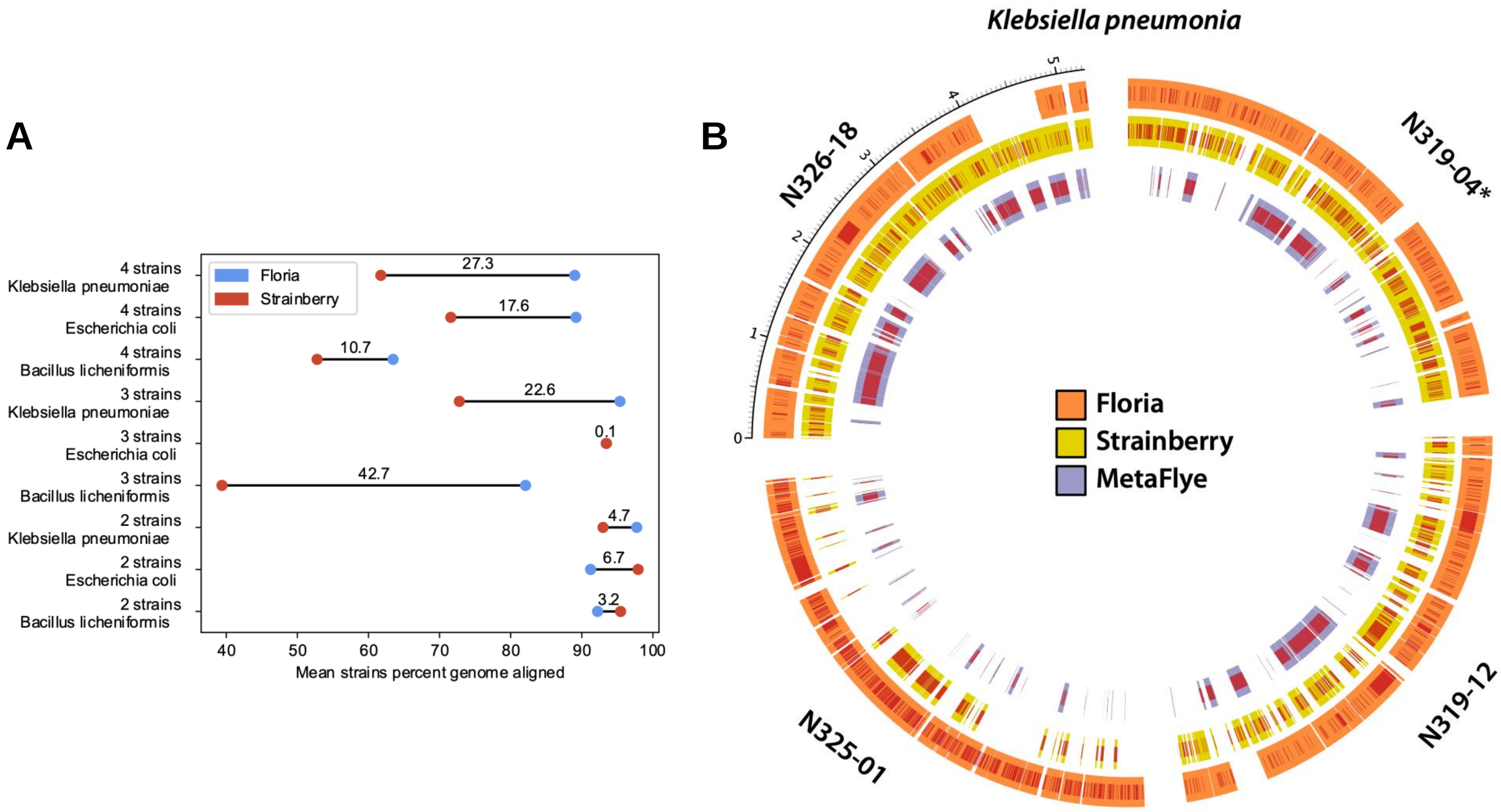

Supplementary Figure 10. Phasing real reads for three different species and varying numbers of strains. A. Mean values of the percent aligned for each multi-strain community, for both Floria (with Flye) and Strainberry. Numbers between dots represent the absolute difference. B. Circos plot of a *Klebsiella pneumonia* 4-strain community. Bands represent aligned sections for each strain genomes and red lines SNPs. Asterisk indicates the reference genome used during the mapping process.

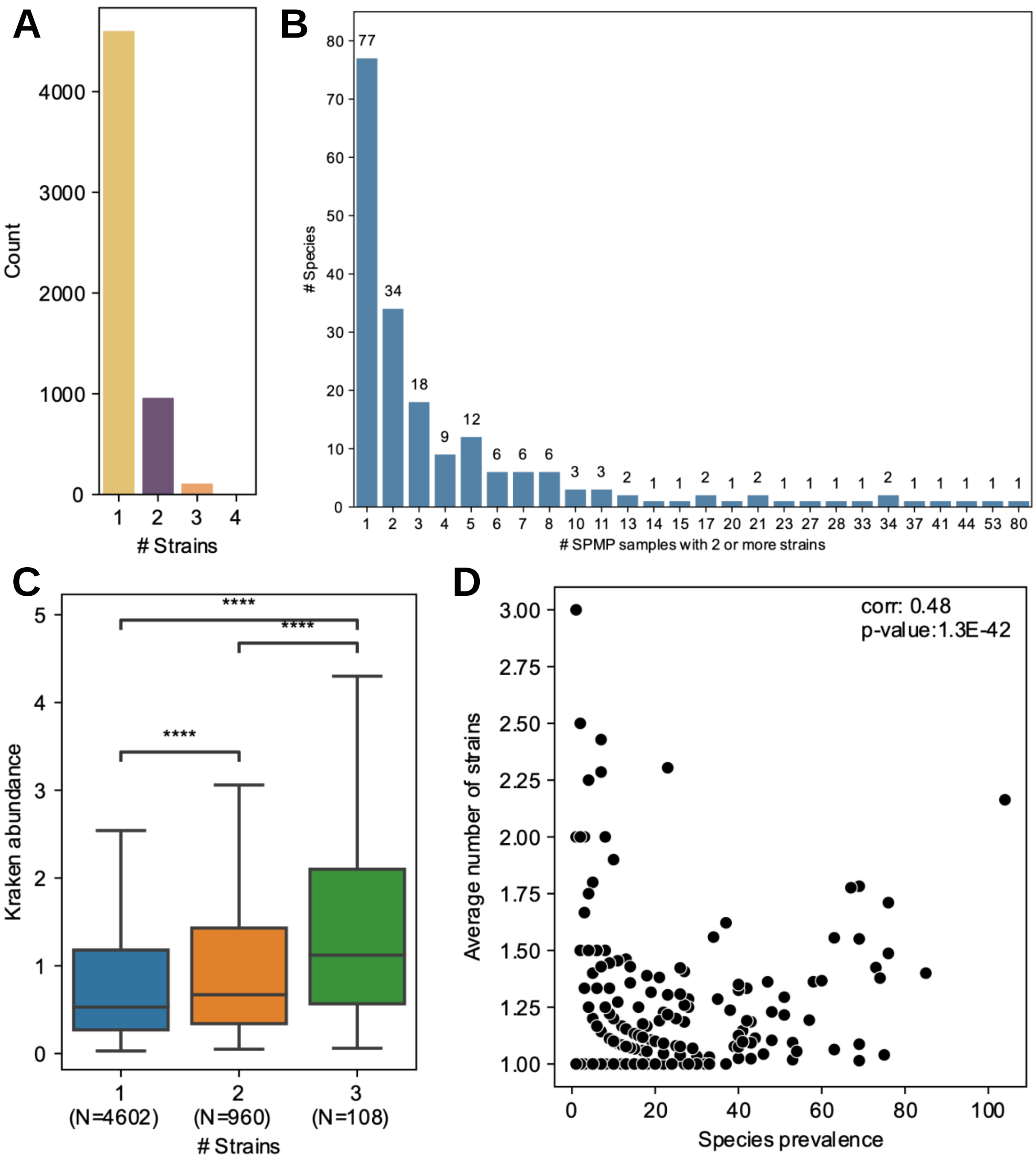

Supplementary Figure 11. SPMP strains phasing results. A. Distribution of the number of strains determined within each species. B. Species with more than 1 strain in one sample are more likely to show similar pattern in other samples. C&D. Number of species correlated with species abundance [C] and prevalence [D]. Flyers are ignored in the boxplot representation. Stars (*) represent significative changes (Mann-Whitney-U test). Associated spearman correlation value and p-value [D]. Effect size (Cohen'D) = 0.086, describing a small effect size.

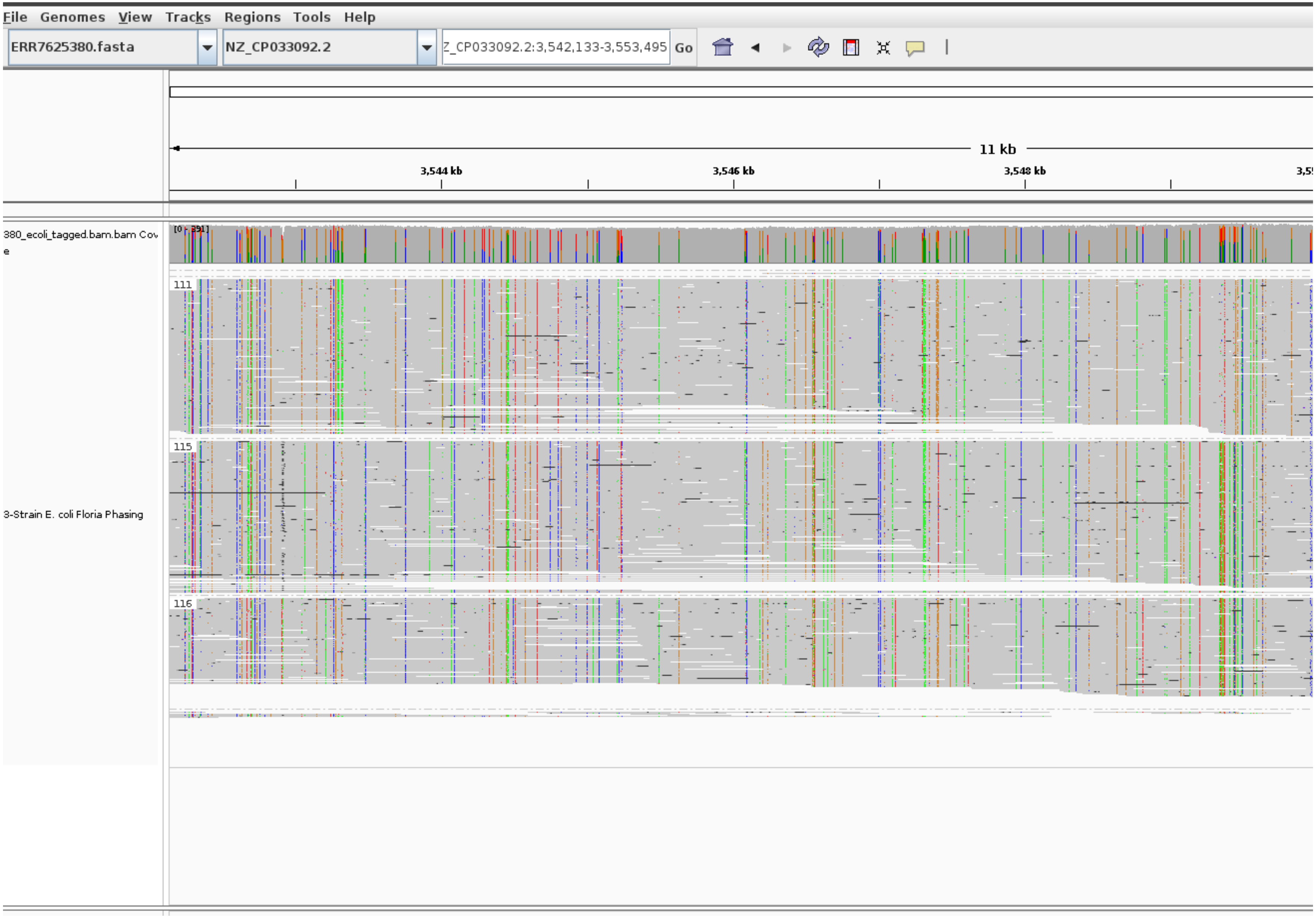

Supplemental Figure 12: Haplosets of *E. coli* for sample ERR7625380 from the SPMP cohort. Three haplosets (labelled 111, 115, and 116) are seen in this section corresponding to an estimated three strains being present.

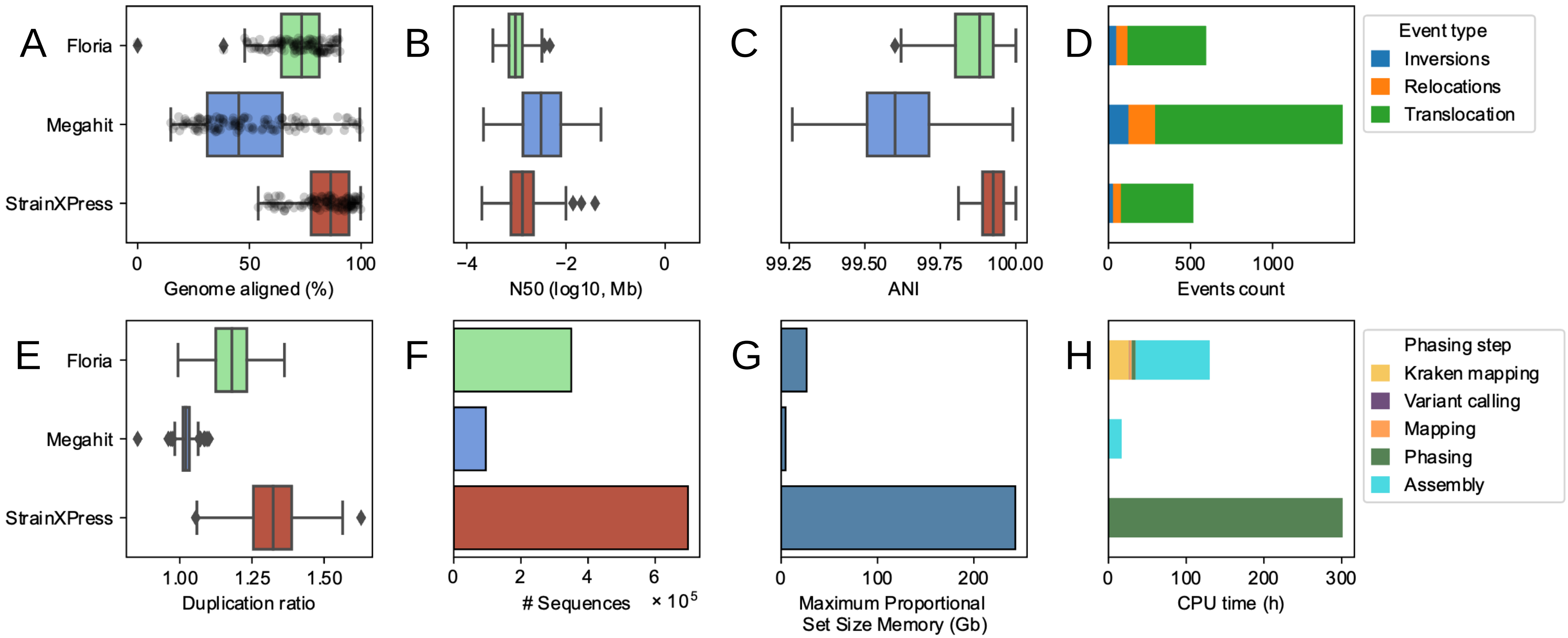

Supplementary Figure 13. Illumina phasing assessment results. Top left to bottom right: (A) Percentage of strains' genomes aligned. (B) N50 of resulting assemblies. (C) ANI of each strain assembly vs. the reference sequence of the strain (RSS). (D&E) Number of structural variants observed (D) and duplication ratio (E) of each phased assembly compared to their respective RSS. (F) Number of contigs obtained for each strain assembly. (G) Maximum memory size reached at any point of the pipeline for each phasing method, adjusted (proportionally) to consider shared library. (H) CPU time in hours for each phasing solution at different steps.

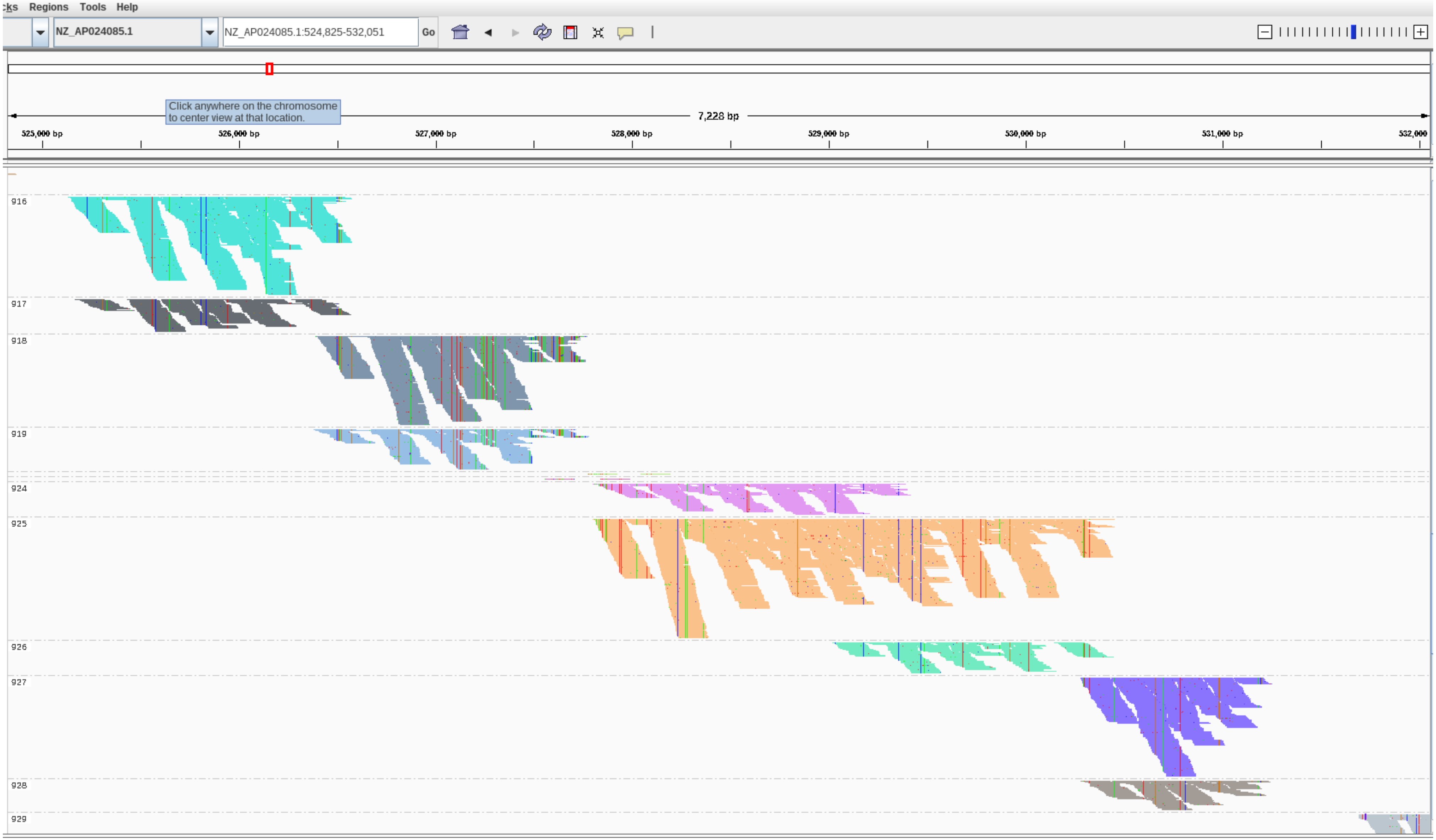

Supplemental Figure 14: Haplosets of NZ_AP024085.1, *Faecalibacillus intestinalis*, output by Floria and visualized for the sample dated at day 0 (in Figure 4) with IGV. Each colored set of mapped reads is a different haploset. Visual inspection shows that for most bases (i.e., the x-axis), exactly two sets of reads are covering it, suggesting the presence of two strains.

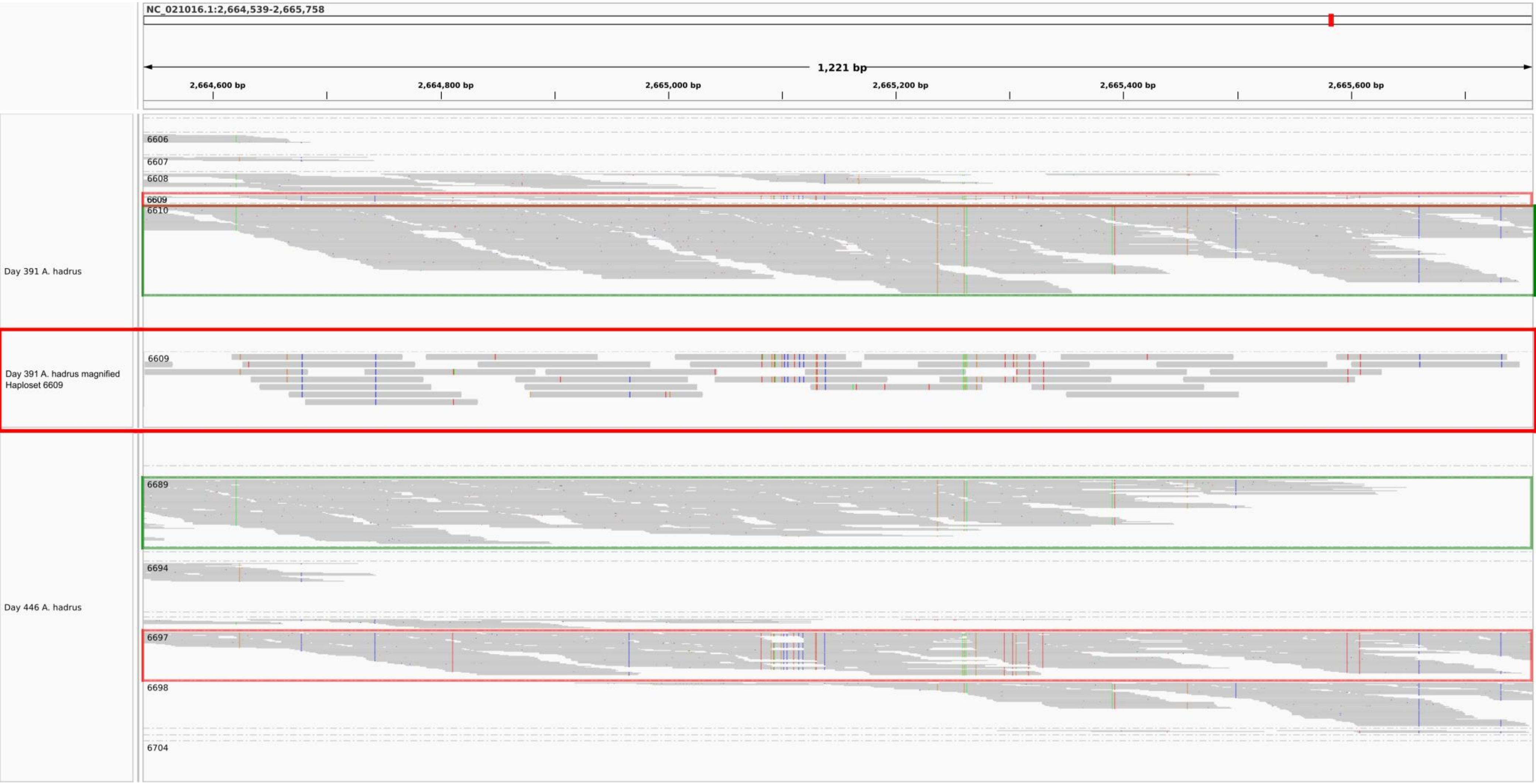

Supplementary Figure 15. Haplosets of NC_021016.1, *Anaerostipes hadrus*, output by Floria and visualized with IGV for the sample dated at days 391 and 446. The boxes with the same color indicate haplosets that share the same SNPs across timepoints. Note that the red box (haploset 6609) at day 391 is at very low coverage and zooming in confirms that this is the emergent strain from Main Figure 4.

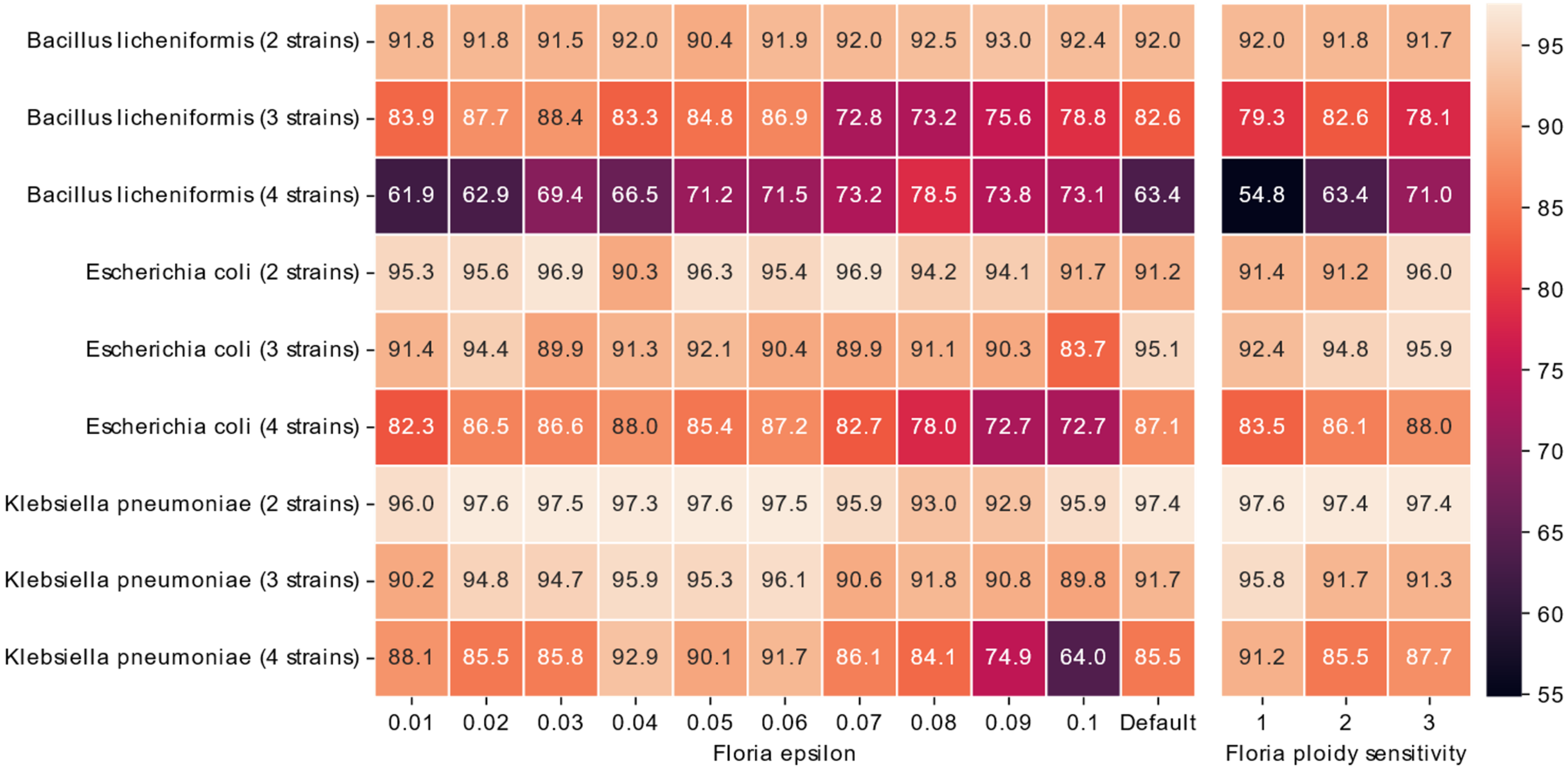

Supplementary Figure 16. **Impact of Floria parameters on phasing results**. Average percent aligned with Floria against the 3 communities with real reads (Figure 2H) and different Floria parameters (epsilon and --ploidy-sensitivity, default = 2). Longshot and Flye for haplotype assembly were used in this analysis. For epsilon evaluation, the default option (epsilon value not provided) leads to an estimation of epsilon by Floria based on read data (see Methods).

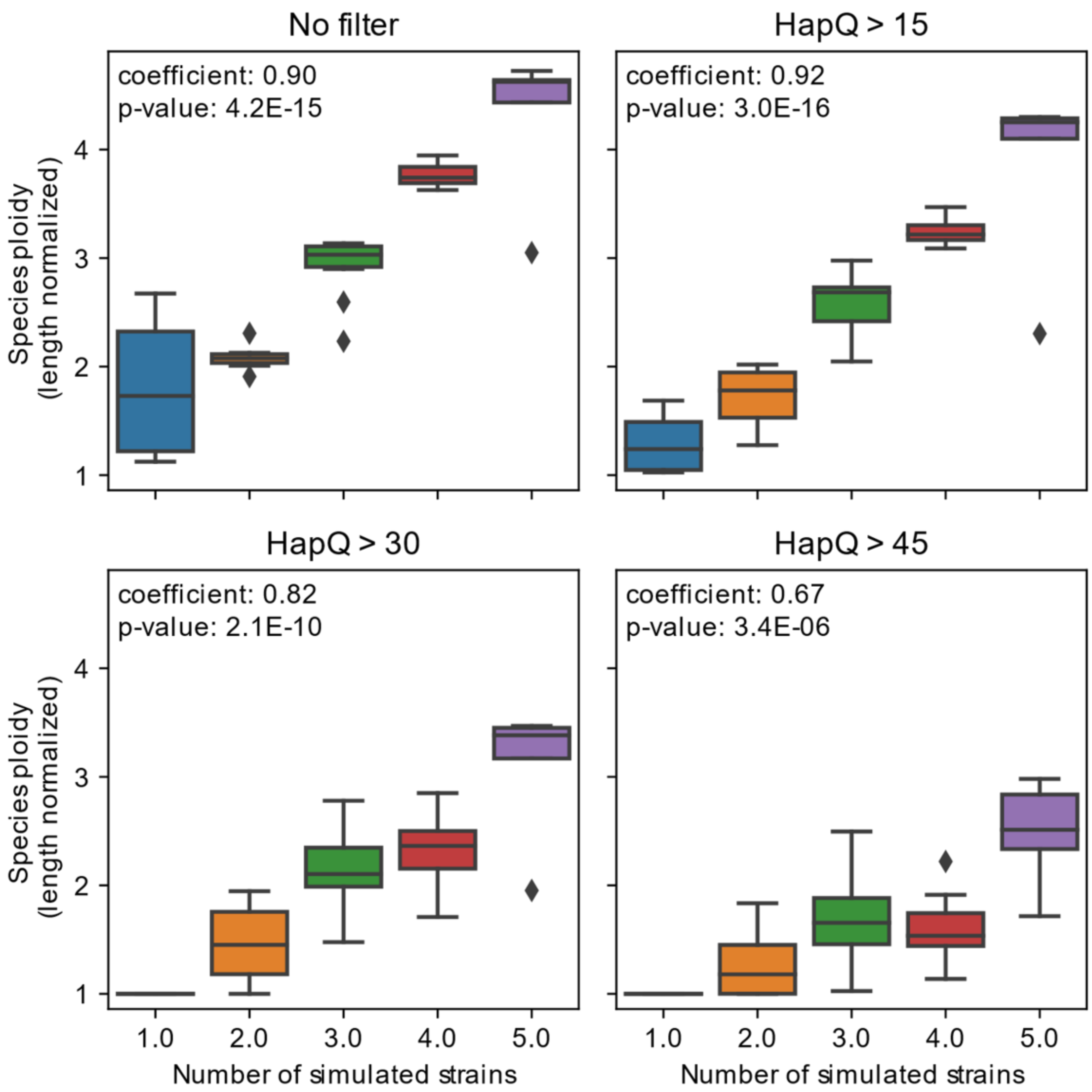

Supplementary Figure 17. **Species ploidy (strain count) estimations for the 40-species synthetic community**. Comparison of the estimated species-ploidy against the number of simulated strains, considering different minimum HAPQ threshold values. The Spearman correlation coefficient with associated p-value is annotated on the top left of each plot.
